# G-quadruplex formation in double strand DNA probed by NMM and CV fluorescence

**DOI:** 10.1093/nar/gkv749

**Published:** 2015-07-21

**Authors:** Alex Kreig, Jacob Calvert, Janet Sanoica, Emily Cullum, Ramreddy Tipanna, Sua Myong

**Affiliations:** 1 Bioengineering Department, University of Illinois; 1304 W. Springfield Ave. Urbana, IL 61801, USA; 2 Biophysics and Computational Biology; 1110 W. Green St. Urbana, IL 61801, USA; 3 Institute for Genomic Biology; 1206 Gregory Drive, Urbana, IL 61801, USA; 4 Physics Frontier Center (Center of Physics for Living Cells), University of Illinois; 1110 W. Green St. Urbana, IL 61801, USA

## Abstract

G-quadruplexes (GQs) are alternative DNA secondary structures that can form throughout the human genome and control the replication and transcription of important regulatory genes. Here, we established an ensemble fluorescence assay by employing two GQ-interacting compounds, *N*-methyl mesoporphyrin IX (NMM) and Crystal Violet (CV). This enables quantitative measurement of the GQ folding propensity and conformation specificity in both single strand (ss) and double strand (ds) DNA. Our GQ mapping indicates that the likelihood of GQ formation is substantially diminished in dsDNA, likely due to the competition from the Watson–Crick base pairing. Unlike GQ folding sequence in ssDNA which forms both parallel and antiparallel GQs, dsDNA displays only parallel folding. Additionally, we employed single molecule FRET to obtain a direct quantitation of stably formed-, weakly folded and unfolded GQ conformations. The findings of this study and the method developed here will enable identifying and classifying potential GQ-forming sequences in human genome.

## INTRODUCTION

The G-quadruplex (GQ) is a four-stranded secondary structure of DNA that arises from a Guanine (G)-rich sequence. The G-rich, single stranded DNA sequences have been shown to fold into stable GQ structures following the basic algorithm of [G_3_N_1–7_G_3_N_1–7_G_3_N_1–7_ G_3_] where triplet G bases are separated by a loop sequence, N (1). This structure is stabilized by Hoogsteen base pairing between guanine bases as well as monovalent cations such as potassium or sodium (2,3). Depending on the presence of specific cations and loop sequence composition, GQ DNA can form into parallel, antiparallel and a mixed hybrid conformations (4–10). Recent studies have drawn increased attention to this structure due to its potential role in regulating biological pathways including transcription and replication (11–14). Due to GQ-specific sequence requirements, these sequences have been a prominent target for bioinformatic studies (15–18). The GQ-forming sequences are unevenly dispersed throughout the human genome, with specific sites including telomeric overhangs, immunoglobulin switch regions and gene regulatory sequences (19–21). Additionally, stable GQs may arise from the hexanucleotide repeat expansion (HRE), (GGGGCC)_n_, which is the most prevalent genetic cause of neuro-degenerative diseases including amyotrophic lateral sclerosis (ALS) and frontotemporal dementia (FTD) (22,23).

Promoters in eukayrotes are enriched with sequences capable of forming GQ structures. Computational studies suggest that GQs are 230 times more likely to be found in promoter regions as compared to the rest of the genome (24). Despite the plethora of reports on telomeric DNA, relatively few studies have looked into GQs in promoters. Furthermore, most studies focused on several well-characterized sequences such as CMYC, TERT and BCL2 formed in the context of ssDNA (25–29). Although ssDNA may be relevant for studying telomeric overhang, it cannot be an appropriate platform for investigating 400 000 potential GQ-forming sequences in double strand DNA found throughout the human genome (24). Thus, we sought to probe GQ formation within a duplexed DNA, which is the native context for the genomic DNA.

We established a method for testing the GQ formation in both ss- and dsDNA. It is a solution-based ensemble fluorescence assay that uses two GQ ligands, *N*-methyl mesoporphyrin IX (NMM) and Crystal Violet (CV). The NMM and CV fluorescence occurs upon binding parallel and antiparallel GQ, respectively. Therefore, the dual-color fluorescence measurement enables detection of GQ formation and the predominant conformation of the folded GQ. Using this method, we mapped a series of potential GQ-forming sequences in both the ss- and dsDNA contexts. Our study reveals that GQ-forming sequences have a dramatically diminished folding propensity in dsDNA compared to ssDNA. We show that the only sequences composed of extremely short loop in all three intervening (non-G) positions, such as CMYC, can fold into a GQ in dsDNA whereas sequences even with slightly longer loop fail to fold in dsDNA. By employing single molecule FRET detection, we provide quantitative analysis of GQ folding, which complements and confirms the results of the ensemble fluorescence assay. We also present dynamic GQ structure that undergoes transient transitions to an unfolded state. The GQ-forming propensity and stability in duplexed DNA we present here may have implications for processes that occur in the context of genomic DNA.

## MATERIALS AND METHODS

### Preparation of DNA

For bulk experiments, GQ DNA sequences and their complements were purchased unmodified from Integrated DNA Technologies (IDT). Single stranded DNA studies were conducted with an 18 mer ssDNA on the 5′ end, while duplexed DNA studies contained two separate 18 mer on both the 3′ and 5′ ends. For single molecule experiments, the same sequences as above were purchased containing an amine-modified thymine located 3 or 4 bases from the GQ-forming region. Constructs were labeled by incubating 10 mM CY3 or Cy5-NHS ester (GE Lifesciences) with .1 mM DNA in 100 mM sodium bicarbonate pH 8.5 buffer for 4–5 h. Excess dye was removed through two rounds of ethanol precipitation. Sequences were diluted in a standard G-quadruplex formation buffer: 20 mM Tris-HCl pH 7.5, 100 mM KCl.

### Annealing dsDNA

Complementary DNA pairs were annealed at 1:1 ratio for ensemble fluorescence measurements. 40% (v/v) PEG 200 (Sigma Aldrich) was supplemented to the standard GQ buffer to induce GQ formation in duplex constructs. The annealing reaction was performed by incubating samples at 95°C in standard GQ buffer for 5 min and then cooling 2°C per min to room temperature (24 ± 1°C). FRET constructs for single molecule imaging were annealed by mixing the Cy5-labeled 3′-biotinylated DNA and complementary 3′Cy3 GQ-containing DNA at a molar ratio of 1:1.2. The constructs were incubated at 95°C for 5 min and then cooled 2°C per min to room temperature (24 ± 1°C). Excess Cy3 labeled oligonucleotide is added to maximize the likelihood of immobilizing duplex on NeutrAvidin coated surface. The excess non-biotinylated strand is removed from the imaging chamber by flushing the system during single molecule FRET imaging.).

### Fluorescence measurements of GQ-ligand interaction

1 μM NMM (Frontier Scientific) and 1.2 μM CV (Sigma Aldrich) were separately mixed with 400 nM dsDNA samples in standard GQ buffer. Final imaging conditions contained 4% PEG 200 (v/v). Control samples that was annealed in non-PEG buffer was tested by adding 4% PEG 200. Lack of fluorescence in these samples indicated that the presence of PEG 200 post annealing would not significantly alter fluorescence measurements or induced GQ folding after the annealing process. Emission measurements were taken with a fluorescence spectrophotometer (Cary Eclipse, Varian) exciting NMM and CV at wavelengths of 393 nm and 540 nm, respectively. Measurements were repeated in buffer containing 5 mM MgCl2 instead of monovalent cations, to ensure changes in emission intensity were the result of ligand binding to folded dsDNA GQs.

### Single-molecule imaging buffer

For single molecule imaging, the standard GQ buffer was supplemented with an oxygen scavenging system of 0.8 mg/ml glucose oxidase, 0.625% glucose, 3 mM 6-hydroxy-2,5,7,8-tetramethylchroman-2-carboxylic acid (Trolox) and 0.03 mg/ml catalase.

### Single-molecule imaging

Single-molecule fluorescence experiments were performed in channels made from glass coverslips on quartz slides (Finkenbeiner). Slides and coverslips were pretreated with methanol, acetone, potassium hydroxide and flame treatment, followed by an aminosilane coating. To prevent DNA–surface interactions, slides and coverslips were coated with 97% methyl-PEG (m-PEG-5000, Laysan Bio, Inc.) and 3% biotin PEG (biotin-PEG-5000, Laysan Bio, Inc). Biotinylated DNA molecules were immobilized to the slide surface through biotin-neutravidin interactions. Imaging buffer was flowed through the chamber to wash out unbound molecules and remove residual PEG 200. All experiments and measurements were performed at room temperature (24 ± 1°C). Total internal reflection microscopy was used to collect single-molecule FRET data. Specifically, an evanescent field of illumination was created by directing a 532-nm Nd:YAG laser through a prism. Signals were collected by a water-immersed objective with a 550 nm long pass filter to remove the scattered light. Donor dye signals were collected using a 630 nm dichroic mirror and a charge-coupled device camera.

### Single molecule data analysis

Data were recorded with a 100 ms time resolution and analyzed with Interactive Data Language (IDL) to give single-molecule traces of fluorescence intensity over time. Data output from IDL was processed with custom MATLAB scripts, which are available to download from https://physics.illinois.edu/cplc/software/. Efficiency of FRET was calculated as the acceptor channel intensity divided by the sum of donor and acceptor channel intensities. Folding populations were calculated through the removal of donor only (Cy3) containing traces and Gaussian fitting the peaks of FRET histograms generated from 20 field of view.

### Circular dichroism

Circular dichroism was performed at room temperature (23 ±1°C) with 10 μM GQ oligonucleotides. Standard GQ-forming buffer, mentioned above, was utilized for annealing and measurement. For CD measurements within the duplex constructs, sequences were annealed at 100 uM in standard buffer supplemented with 40% (v/v) PEG 200. The CD spectra were recorded on a JASCO J-715 spectropolarimeter over the range of 200–320 nm using a 1-mm path length quartz cuvette with a reaction volume of 200 μl.

**Figure 1.**
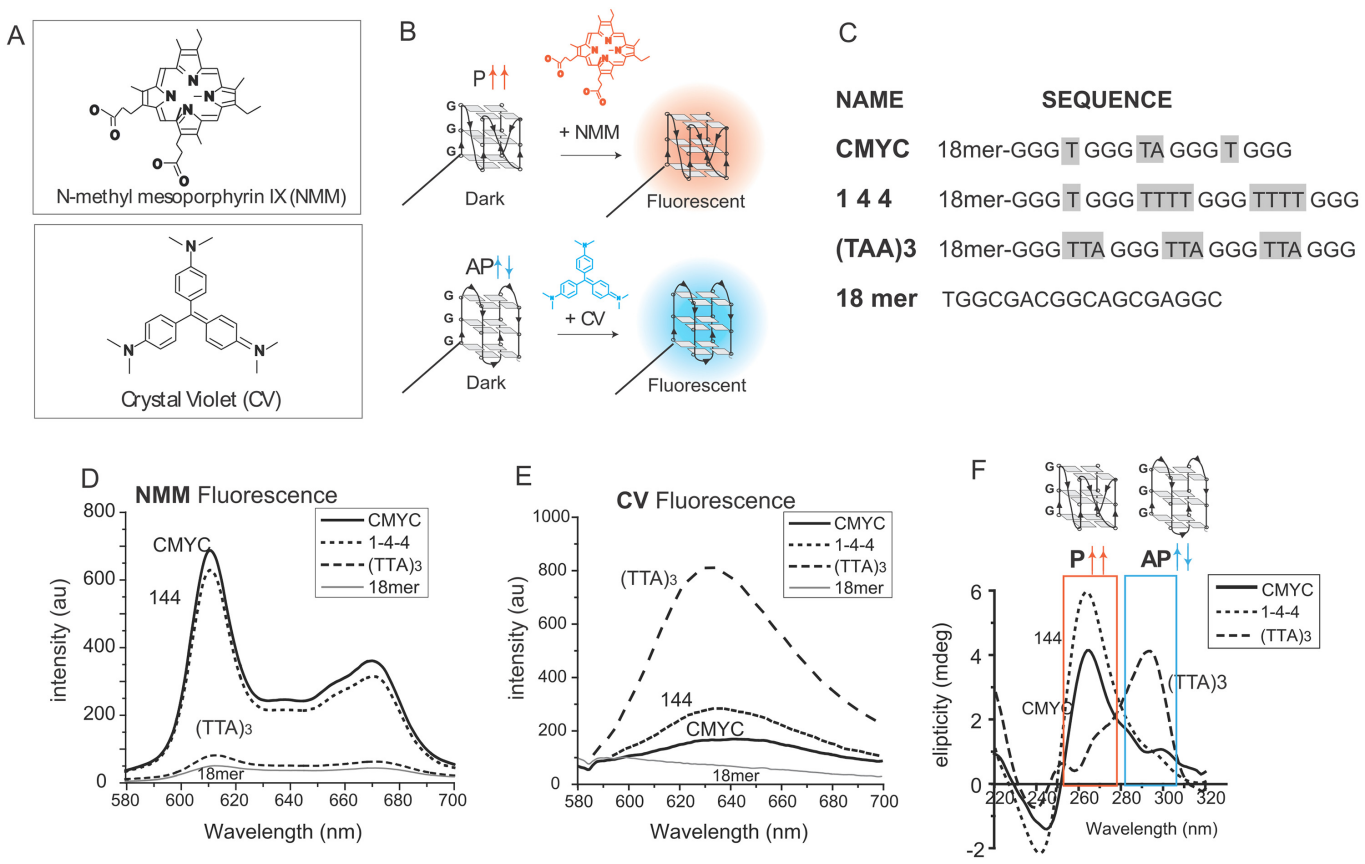
NMM and CV binding to GQ DNA. (**A**) Chemical structure of NMM and CV. (**B**) Schematic of fluorescence assay by using NMM and CV. (**C**) DNA sequence of three constructs used here. (**D, E**) Scanned emission spectrum of NMM (D) and CV (E) bound to four DNAs. (**F**) Circular dichroism spectrum for three GQ-forming DNAs.

For GQ in ssDNA presented in Figure 1F, we took CD spectra of all four DNAs, CMYC, 1–1–1, (TTA)_3_ and 18mer and subtracted the CD spectra of 18mer from the three GQ-forming DNAs. For GQ in dsDNA presented in Supplementary Figure S4, we took CD spectra of all CMYC, 1–1–1, (TTA)_3_ duplex DNAs and subtracted the CD spectra of 18 base pairs corresponding to one of the duplex handles attached to each DNA construct.

## RESULTS

### NMM and CV fluorescence measures GQ folding and distinguishes GQ conformation

Previously, we reported two ways of measuring GQ conformation in ssDNA. SmFRET was used in conjunction with circular dichroism (CD) to quantitatively measure and verify the GQ conformations. Additionally, we developed NMM quenching assay in which NMM binding to Cy3 labeled GQ DNA quenches the Cy3 fluorescence. The results clearly indicated that NMM binding and concomitant quenching is specific to parallel GQs (10). While these assays are useful for quantitative measurement of parallel GQs, both the FRET and quenching assays require fluorescence tagging of DNA, which can be costly and inefficient. Therefore, we set out to develop an assay that can be performed on unlabeled DNA in both ss- and dsDNA. Such approach can be useful for high-throughput screening of genomic DNA.

The assay relies upon the induced fluorescence NMM and CV upon binding GQ DNA (30–33) (Figure 1A). NMM and CV exhibit conformation-dependent binding and fluorescence to parallel and antiparallel GQs, respectively (34,35) (Figure 1B). We leveraged this difference to determine the dominant folding motif of GQ-forming DNA with varying loop lengths. To test the feasibility of discerning GQ conformation by NMM and CV, we selected three previously-characterized GQ-forming sequences, CMYC (parallel GQ), 1–4–4 (parallel GQ) and (TTA)_3_ (primarily antiparallel GQ) in ssDNA (10). We named the sequences after their loop length or composition. For example, 1–4–4 is GGG T GGG TTTT GGG TTTT GGG and (TTA)3 is GGG TTA GGG TTA GGG TTA GGG (Supplementary Table S1). These sequences were compared to a non-GQ-forming negative control, 18mer (Figure 1C). In order to promote GQ folding, we used buffer containing 100 mM potassium chloride (KCl) in all measurements. When NMM (1 μM) was applied to individual DNA (400nM) sample, both CMYC and 1–4–4 exhibited a high level of fluorescence The two emission peaks at 610 and 670 nm represent fluorescence of NMM induced by parallel GQ binding. In contrast, (TTA)_3_ showed substantially reduced 610 and 670 nm fluorescence peaks, indicating diminished parallel conformation. This is consistent with low level of induced fluorescence of NMM caused by antiparallel structures (36). The 18-mer control showed the lowest fluorescence values, indicating a negligible level of nonspecific binding of NMM to non-GQ-forming DNA (Figure 1D). The high NMM fluorescence displayed for CMYC and 1–4–4, and the low level for (TTA)_3_ are in agreement with our previous study in which we reported highly-parallel folding of CMYC and 1–4–4, and primarily non-parallel folding of (TTA)_3_ by FRET analysis (10). We confirmed that neither NMM nor CV exhibits fluorescence in the absence of GQ-forming DNA (Supplementary Figure S1A).

Using the same set of DNAs, we performed the assay with CV (1.2 μM), the marker ligand for antiparallel GQ. Contrary to the pattern observed for NMM, CV yielded the highest fluorescence for (TTA)_3_ and the lowest intensity for CMYC and 1–4–4 (Figure 1E). This result confirms the dominant antiparallel GQ folding of (TTA)_3_ and lack of antiparallel conformation in CMYC and 1–4–4. We note that the (TTA)_3_ may also form into hybrid state, but CV fluorescence only reports on the presence of antiparallel GQ. Again, the 18-mer showed a near-zero fluorescence level, which indicates minimal non-specific binding of CV to non-GQ DNA. Furthermore, CD measurements confirm that CMYC and 1–4–4 fold in a parallel conformation, as displayed by the distinct peak at 260 nm and a valley at 240 nm (37–39). In contrast, (TTA)_3_ primarily folds in antiparallel (34), as indicated by a major peak at 290 nm (Figure 1F). Here, we demonstrate the feasibility of employing the NMM and CV for measuring GQ folding and distinguishing GQ conformations.

### Applying NMM and CV to map GQ-forming sequences

**Figure 2.**
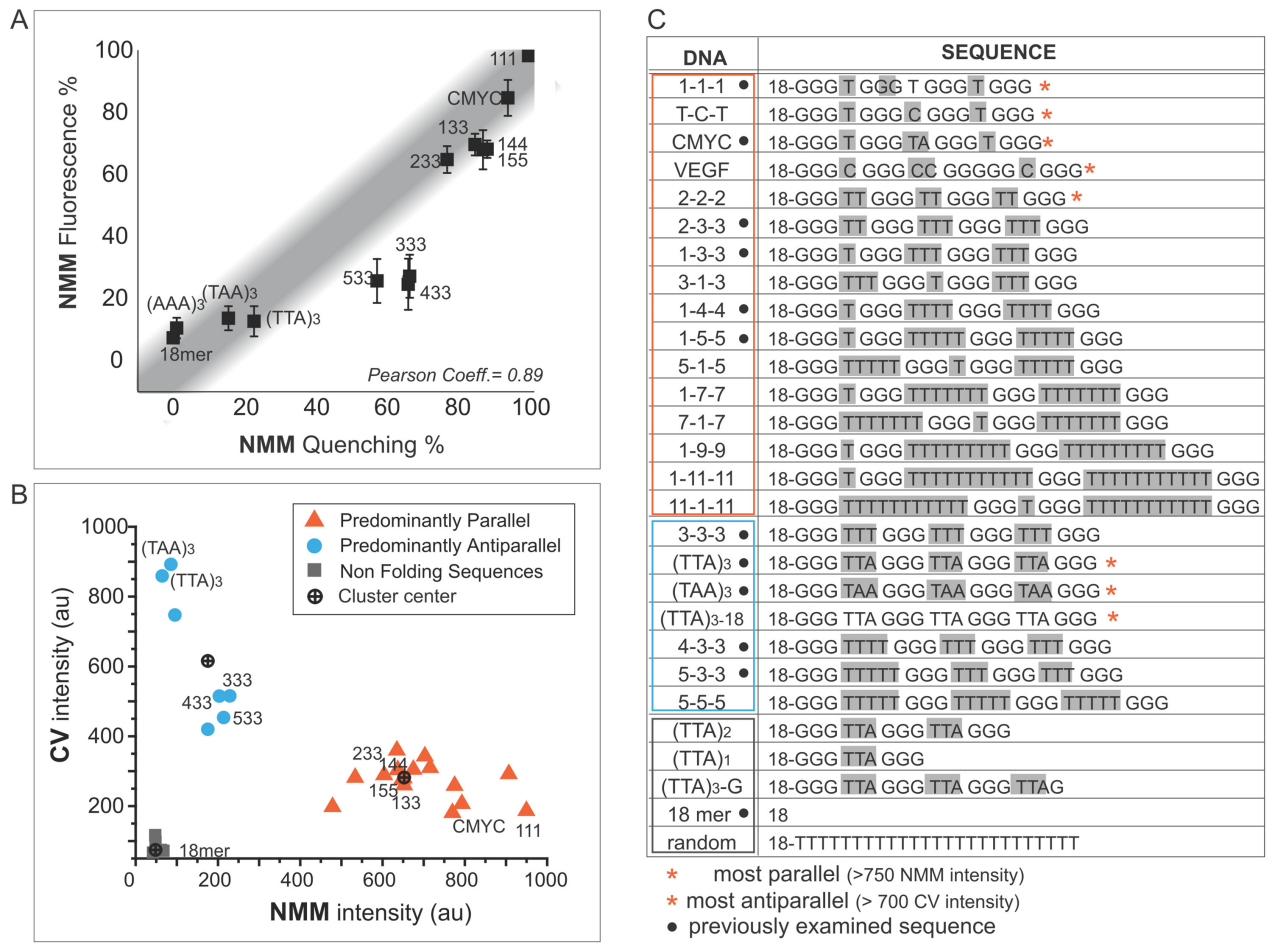
NMM and CV fluorescence induced by GQ-forming DNAs. (**A**) Comparison of NMM quenching (previously published) and NMM fluorescence. (**B**) Scatter plot of NMM and CV fluorescence for all DNAs show three clusters corresponding to parallel, antiparallel and unfolded groups. (**C**) All DNA sequences used for NMM and CV fluorescence measurement.

We chose a set of 12 GQ-forming sequences to further evaluate the NMM fluorescence assay by comparing it to the NMM quenching data obtained previously. Peak value of fluorescence induced by NMM (610 nm) was plotted against the NMM quenching data reported previously (10). As shown, the two data sets were highly correlated as shown by the scatter plot (Pearson coefficient of 0.89), validating the NMM fluorescence method for measuring the presence of parallel GQ (Figure 2A). In both the% quenching and% fluorescence data, the value of 1–1–1 was set as 100%. The slightly lower fluorescence value obtained for 3–3–3, 4–3–3 and 5–3–3 is likely due to the fast dynamics exhibited by these DNA substrates, which may reduce the fluorescence of NMM (10). Briefly, our smFRET measurement on 3–3–3, 4–3–3 and 5–3–3 showed that these DNAs undergo fast transitions to unfolded state, parallel and non-parallel states. Such conformational dynamics may have reduced the emission output by NMM. In both sets of data, the emerging pattern is that short loop promotes parallel GQ folding while longer loop results in less parallel structures and unfolded state.

Having tested the validity and feasibility of the NMM and CV fluorescence method, we measured both NMM (610 nm) and CV (640 nm) fluorescence for a larger set of twenty three DNAs listed in Figure 2C and Supplementary Table S1. To represent the two sets of data in a single view, the fluorescence values of NMM and CV obtained for all DNAs were used to build a scatter plot where the *x*-axis and *y*-axis are NMM and CV intensity, respectively (Figure 2B). It displays three clearly distinguished clusters of sequences, indicating three separate GQ conformations. First, the orange triangles represent the parallel species including CMYC, 1–3–3 and 1–4–4 that exhibit high NMM and low CV induced fluorescence. Second, the light blue circles indicate less parallel and more antiparallel GQs including (TAA)_3_ and (TTA)_3_ that display high CV coupled with low NMM intensity. These sequences exhibited primarily antiparallel folding in our previous study, confirming the CV fluorescence as a measure of antiparallel conformation (10). Third, the gray squares are the unfolded DNAs to which neither NMM nor CV bind. The center of each cluster is marked with a crosshair. NMM alone is sufficient for measuring the parallel state of a GQ, while CV provides a complementary index of antiparallel GQs. Together, our results demonstrate that dual color ensemble assay utilizing NMM and CV fluorescence can be a reliable measurement for mapping GQ-forming propensity and conformation.

Based on the results shown in Figure 2A and B, the sequences in Figure 2C are organized into three distinct populations. The sequences in orange box are predominantly parallel whereas the sequences in light blue box are primarily antiparallel folding sequences. Non-folding sequences are located within the gray outline. As previously shown, folding conformation is dependent of the loop length (10). Increasing the loop length results in transition from predominantly parallel to antiparallel and unfolded state.

### GQ formation in double-stranded DNA

Promoter DNA that harbors GQ-forming sequences occurs in the context of duplexed DNA. It is predicted that this segment of DNA will typically exist as dsDNA, indiscriminant from other genomic DNA. It is thought that at the initiation of transcription, DNA is unwound by transcription machinery, generating a negative supercoiling and stimulating the formation of GQs (40). This formation of GQs may serve to dissipate the torsional strain built-up in the helical structure of DNA. Previous biochemical study showed that GQ formation can be induced by annealing the G-rich strand and the complementary C-rich strand in the presence of the molecular crowding reagent, Polyethylene Glycol (PEG) (41). This annealing method to form GQs in dsDNA allows one to emulate the transcription-triggered GQ formation. Adopting this protocol, we annealed DNA in the standard potassium ion concentration with the addition of 40% PEG. For fluorescence measurements, the DNA was diluted tenfold, resulting in 4% PEG.

**Figure 3.**
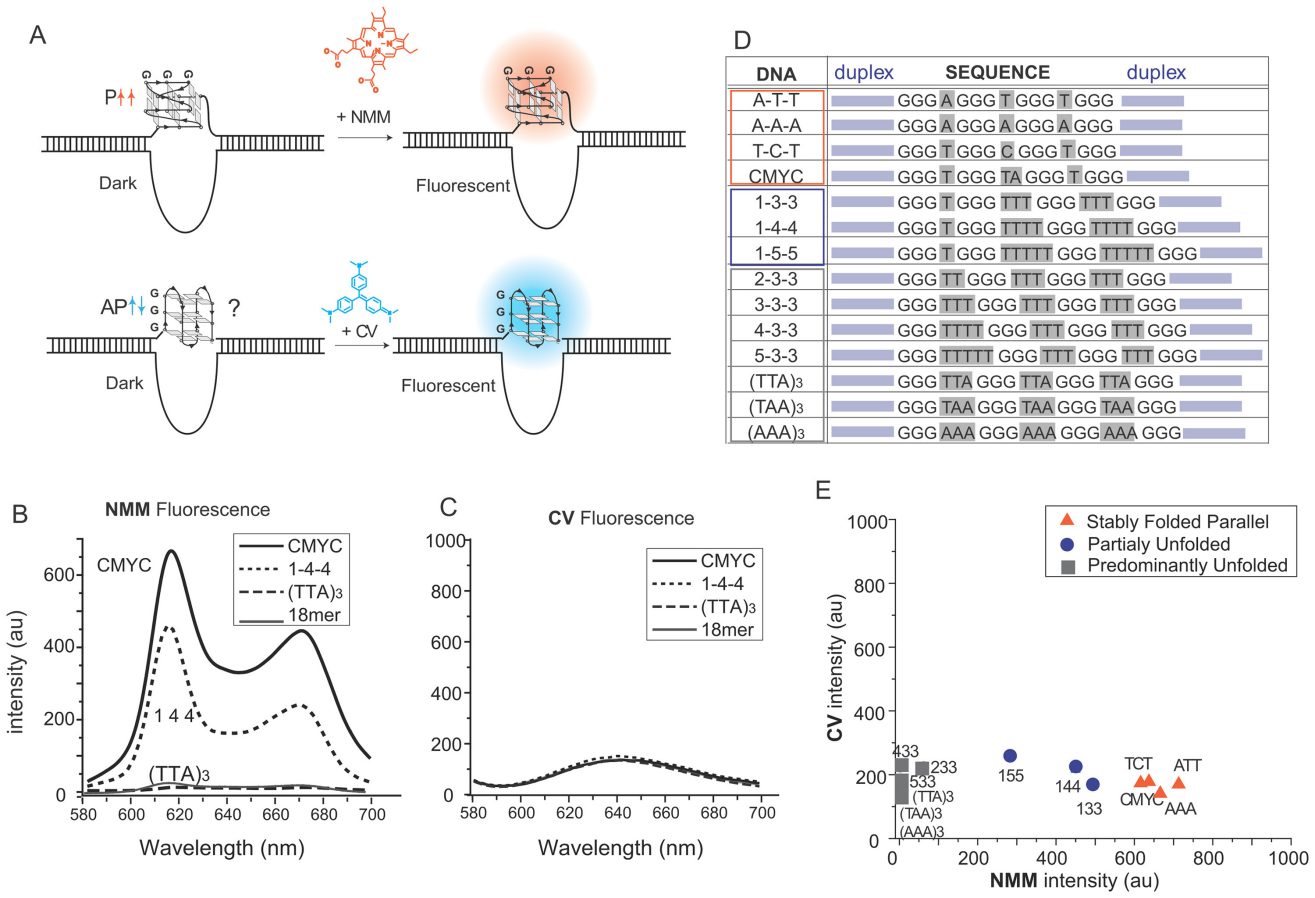
NMM and CV fluorescence assays on GQ formed on double strand DNA. (**A**) Schematic of NMM and CV binding to GQ on dsDNA. (**B, C**) Scanned emission spectrum of NMM (B) and CV (C) for the four DNAs. (**D**) All DNA sequences used for GQ formation in dsDNA. (**E**) Scatter plot of NMM versus CV fluorescence.

The dual-color fluorescence assay was performed for the GQ formed in dsDNA using the same protocol used for testing GQ in ssDNA (Figure 3A). When annealed in this condition (40% PEG), the double-stranded (ds) CMYC showed the highest fluorescence upon addition of NMM, followed by 1–4–4, suggesting that GQ formation occurred in both DNAs. Unlike in ssDNA, where the two DNAs displayed the same level of fluorescence, the NMM fluorescence for 1–4–4 is substantially lower than that of CMYC, suggesting a less efficient GQ folding of 1–4–4 than of CMYC in the dsDNA context (Figure 3B). (TTA)_3_ shows no fluorescence, which is indistinguishable from the control 18-mer dsDNA, indicating that (TTA)_3_ does not fold into parallel GQ. When the DNAs are annealed in a buffer devoid of PEG, we observe no fluorescence of NMM and CV, suggesting no formation of GQ in all three DNAs (Supplementary Figure S1B). We also show that the addition of PEG to already annealed DNA duplexes cannot stimulate GQ formation (Supplementary Figure S1C).

In stark contrast to the GQs formed in ssDNA, CV fluorescence yielded uniformly negligible fluorescence for all GQs formed in dsDNA (Figure 3C), indicating that none of the three DNA substrates fold into the antiparallel conformation. In particular, the contrast between the high CV fluorescence of (TTA)_3_ seen in ssDNA (Figure 2C) and no fluorescence in dsDNA clearly suggests that the antiparallel folding only exists in the context of ssDNA. It also implies that antiparallel folding cannot be supported in dsDNA likely due to the competition with Watson–Crick base pairing.

We examined several additional genomic GQ DNA constructs in dsDNA (Figure 3D). In the first group (orange box), the shortest loop of varying sequence composition (A-T-T, A-A-A, T-C-T) were tested in order to compare with CMYC. All three DNAs yielded high NMM fluorescence coupled with low CV fluorescence, both comparable to CMYC, reflecting formation of primarily parallel GQs (Figure 3E). The second group (blue box) includes 1–3–3, 1–4–4 and 1–5–5, all of which are highly parallel in ssDNA (Figure 2B). In duplex, however, all three constructs showed an intermediate level of NMM (1–3–3>1–4–4>1–5–5) coupled with low CV fluorescence, which we later show as arising from a mixed population of duplexed and GQ folded population of molecules. The third group, including 2–3–3, 3–3–3, 4–3–3, 5–3–3, (TTA)_3_, (TAA)_3_ and (AAA)_3_ (gray box) all displayed extremely low NMM and low CV fluorescence. This indicates no formation of GQs within this group. Again, this result starkly contrasts the antiparallel folding exhibited by these sequences in ssDNA (Figure 2C). To check if the CV binding was prevented by the presence of duplex or the complementary C-rich strand, we performed a control experiment in which (TTA)_3_ sequence was annealed with a non-complementary DNA sequence composed of poly thymine (Supplementary Table S1). In this DNA, we expect the formation of antiparallel GQ due to the lack of complementary strand. Upon addition of CV, we obtained a clean fluorescence peak that arises from the formation of antiparallel peak. This result strongly suggests that the lack of CV fluorescence is due to lack of GQ formation, not due to the perturbation by duplex or complementary strand (Supplementary Figure S2). Taken together, GQ formation in dsDNA is significantly less favorable than in ssDNA, likely due to the competition introduced by Watson–Crick base pairing. Despite these inhibitory forces, the highly parallel sequences composed of short loop manage to fold and maintain the folded structure over periods of time ranging up to 45 min (Supplementary Figure S3). CD, the standard method of measuring GQ formation, cannot decipher GQ formation in the context of dsDNA. When we tested CD for CMYC, 1–4–4 and (TTA)_3_ formed in dsDNA, all three DNAs yielded a broad and uniform peak at 275 nm. This is likely arises from CD's sensitivity to many other components in the system including duplex arms on both sides of GQ and possible i-motif on complementary C-rich DNA. Therefore, we conclude that CD measurement cannot measure GQ formation in duplexed DNA (Supplementary Figure S4).

### Single molecule FRET analysis of GQ folding

**Figure 4.**
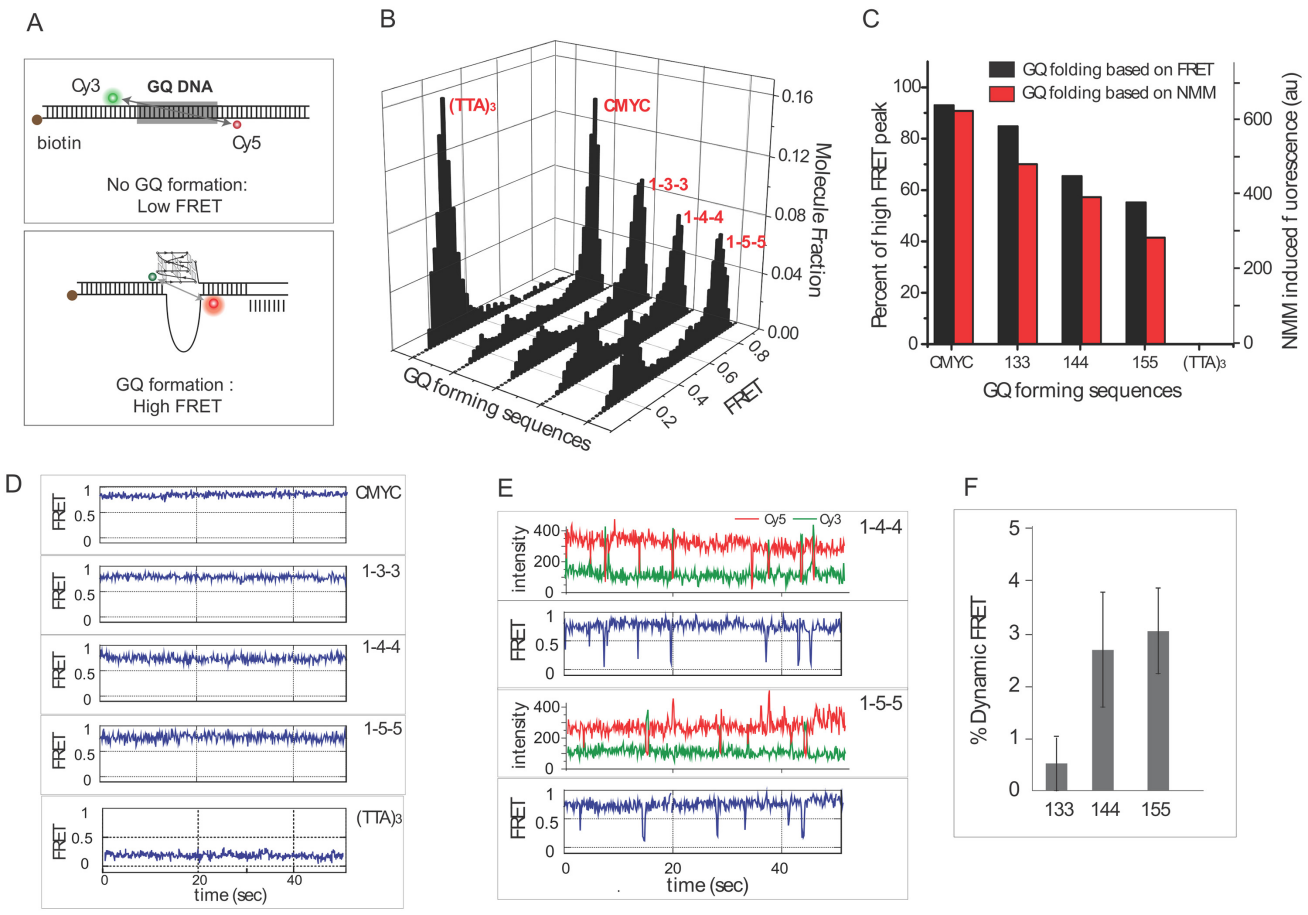
Single molecule FRET assay to visualize GQ formation in dsDNA. (**A**) Schematic of DNA substrate with two dyes, Cy3 (green) and Cy5 (red) located on either side of GQ-forming sequence in dsDNA. (**B**) FRET histograms from five GQ-forming DNA annealed presence of molecular crowding reagent, PEG. (**C**) Quantitation of GQ folding by smFRET (black) and NMM fluorescence (red). (**D**) Representative single molecule trace obtained for five DNAs tested here. (**E**) smFRET traces of 1–4–4 and 1–5–5 exhibiting dynamic FRET fluctuations. (**F**) Quantitation of percent of molecules that display dynamic FRET.

To gain further insight into the GQ folding landscape in dsDNA, we employed single molecule FRET detection. We prepared fluorescently-labeled DNA in which one strand is labeled with Cy3 dye (green) and the complementary strand is labeled with Cy5 dye (red). When annealed, the dyes are expected to be located across the DNA duplex (diagonally) with GQ formed in between (Figure 4A, top). Therefore, the formation of a GQ is expected to induce high FRET due to the reduction in dye-to-dye distance (Figure 4A bottom). The formation of dsDNA without GQ will result in low FRET due to a long distance between the two dyes. We tested five of the previously tested DNAs, CMYC, 1–3–3, 1–4–4, 1–5–5 and (TTA)_3_ by smFRET. We chose these DNAs based on their NMM/CV profiles (Figure 3E) representing highly parallel (CMYC), somewhat parallel (1–3–3, 1–4–4, 1–5–5) and no GQ folding ((TTA)_3_). One end of DNA is biotinylated for immobilization of the DNA onto a single molecule imaging surface coated with NeutrAvidin (42). Once immobilized, one field of view yields approximately 400–500 single molecules of DNA displaying FRET. Typically, we imaged 10–20 fields of view (5–10 s movies) to collect FRET values from over 5000 molecules to generate a FRET histogram. We first tested the sequences annealed in the absence of PEG. As expected, the FRET histogram for all five DNAs yielded uniformly low FRET peaks, consistent with the formation of duplexed DNA (Supplementary Figure S5). The FRET peak for CMYC is slightly higher than the peak for 1–4–4 and (TAA)_3_ because the total length of CMYC (16 bp) is shorter than that of the 1–3–3 (19 bp), 1–4–4 (21 bp), 1–5–5 (23 bp) and (TTA)_3_ (21 bp). We note that the FRET peak values are lower than expected from separation distances of 16 and 21 bp, (∼0.5 and ∼0.275 FRET values), because both dyes are located 3–4 bp away from the GQ-forming sequence boundary. To avoid molecules with only Cy3 fluorophore (donor-only), we selected only molecules that possess both Cy3 (donor) and Cy5 (acceptor) for all FRET histograms. This was achieved by a short pulse of red laser illumination in the beginning of the data acquisition to mark out the molecules that have red dye. In data processing, we can select out the molecules that possess both dyes. Therefore, the low FRET peak arises from the DNA molecules that are duplexed rather than the leakage signal from donor-only molecules.

Next, the DNAs were annealed in 40% PEG-containing buffer and immobilized onto a single molecule imaging surface. After immobilization, we applied PEG-free buffer to remove any PEG-induced effect. In this condition, the FRET peaks for CMYC showed a nearly complete shift to high FRET, consistent with the stable formation of GQ (Figure 4B). This result confirms that the high NMM fluorescence induced by CMYC does not depend on the presence of 4% PEG in the buffer (Figure 3B, E). In the case of 1–3–3, 1–4–4 and 1–5–5, the FRET peak shows two distinct populations. We interpret the high FRET peak (0.8) as GQ folded and a low FRET peak (0.3) as duplexed DNA molecules. We note that the FRET peaks obtained for all five DNAs are highly similar i.e high FRET at 0.8 and low FRET at 0.3. The pattern of FRET distribution between the folded and duplexed DNA for 1–3–3, 1–4–4 and 1–5–5 is in agreement with the intermediate values of NMM fluorescence obtained for this set of DNAs (Figure 3B, E). The quantification of Gaussian fitted area under the high FRET peak is plotted in black bars (Figure 4C). There is a descending pattern of high FRET peak from CMYC (92%), 1–3–3 (86%), 1–4–4 (66%), 1–5–5 (58%) and (TTA)_3_ (0%). This displays that the increasing loop size substantially lowers the propensity for GQ formation in dsDNA. (TTA)_3_ does not exhibit a high FRET peak, in agreement with the negligible NMM fluorescence (Figure 3C, E). This verifies that (TTA)_3_ cannot form into a GQ structure within a dsDNA setting. (Figure 4B, C). To compare the NMM and FRET method for testing GQ formation, we plotted the two sets of data side by side (Figure 4C; black = FRET, red = NMM). The similar pattern between the two data sets confirms that the ensemble fluorescence of NMM allows quantitative measurements of GQ folding status. The lack of non-parallel folding GQs within a duplex construct allows for the use of NMM as the sole GQ probing compound. The same DNA strands annealed in the absence of PEG produced only a low FRET peak corresponding to completely duplexed DNA configuration (Supplementary Figure S5).

Next, we looked into single molecule traces obtained the five DNAs. To obtain long traces, we recorded traces for 1–2 min with 100 ms exposure time. From one movie, we obtain approximately 100–200 single molecule traces that display both dyes without photobleaching for over 1 min. All traces of CMYC exhibit constantly high FRET value whereas all (TTA)_3_ molecules exhibit constant low FRET as expected from the FRET histogram peaks. 1–3–3, 1–4–4 and 1–5–5 show both high FRET (shown) and low FRET traces (not shown), reflecting GQ folded and duplexed DNA populations, respectively (Figure 4D). In addition, 1–4–4 and 1–5–5 DNAs exhibits small percentage (3–5%) of traces that show dynamic FRET fluctuations (Figure 4E, F). Such FRET fluctuations are specific to these DNAs as it is completely absent in CMYC and (TTA)_3_. The unique dynamics exhibited in 1–4–4 and 1–5–5 suggest that the GQ folding is likely less stable than in CMYC, and such weak structure allows it to spring back and forth between folded and partially unfolded conformations even in the context of dsDNA. In contrast, the steady FRET value seen for CMYC indicates that it is tightly locked in a stably folded structure.

## DISCUSSION

We sought to develop a simple and reliable ensemble assay to test the folding and conformational state of GQ in both ss- and dsDNA. We have utilized two GQ-interacting ligands, NMM and CV, which are known to bind selectively to parallel and antiparallel GQs, respectively (34,35). We were able to validate the method by comparing the fluorescence to the previously reported quenching results (10). When the NMM fluorescence assay was applied to the set of sequences previously examined by NMM quenching, CD and smFRET, the result showed high correlation, further validating the use of NMM fluorescence as a GQ folding assessment method (Figure 2A) (10). The distinct fluorescence peaks produced by GQ bound NMM and CV corresponding to the expected conformational state of GQ confirms the validity of this dual-color fluorescence assay.

The NMM fluorescence of 3–3–3, 4–3–3 and 5–3–3 which was slightly lower than the degree of quenching (Figure 2B) is likely due to these DNAs undergoing rapid folding and unfolding transitions as we previously reported (10). While the short-lived interactions between NMM and GQs may be sufficient for quenching of Cy3 dye, the induced fluorescence of NMM may require more stable and long-lived interactions between NMM and GQ structures.

After our initial validation, NMM and CV fluorescence was tested on an expanded list of 23 GQs in ssDNA and five non-GQ-forming negative controls. All 23 GQ-forming sequences showed an anti-correlated pattern (i.e. high NMM coupled with low CV or high CV coupled with low NMM) (Figure 2C). There are two patterns seen in both parallel (orange) and antiparallel (light blue) groups of DNAs. The CV fluorescence for parallel sequences (orange) and NMM fluorescence for antiparallel DNAs (light blue) exhibit a narrow distribution, suggesting a uniformly low binding of the non-matching ligand. In contrast, the fluorescence of the matching ligand, NMM for parallel and CV for antiparallel, displays a broad distribution, representing a wide range of stability in GQ within each conformation. Sequences such as 1–7–7 and 1–9–9 induce lower NMM fluorescence than CMYC, likely due to the presence of the unfolded molecules as shown before (10). As discussed below, such differences become substantially more pronounced in dsDNA (Figure 3E).

It is evident that the length of loop plays a critical role in determining GQ folding and conformation (Figure 2C). In general, longer length promotes antiparallel (non-parallel) and unfolded configurations. Nevertheless, there are clear exceptions in ssDNA, such as 1–5–5, 1–7–7, 1–9–9, 1–11–11 and their permutations, which form primarily parallel GQ. Although, these sequences possess a longer total length of loop than 3–3–3, 4–3–3, 5–3–3, (TTA)_3_ and (TAA)_3_, they do not fold into antiparallel GQ. This is consistent with our previous finding that the presence of a single nucleotide loop triggers parallel folding despite the greater sum of all three loop lengths (10). Based on these results, we conclude that the distribution of the loop length governs GQ folding more than the sum of the total loop length and that one nucleotide loop has a dominant effect in driving the parallel GQ formation.

**Figure 5.**
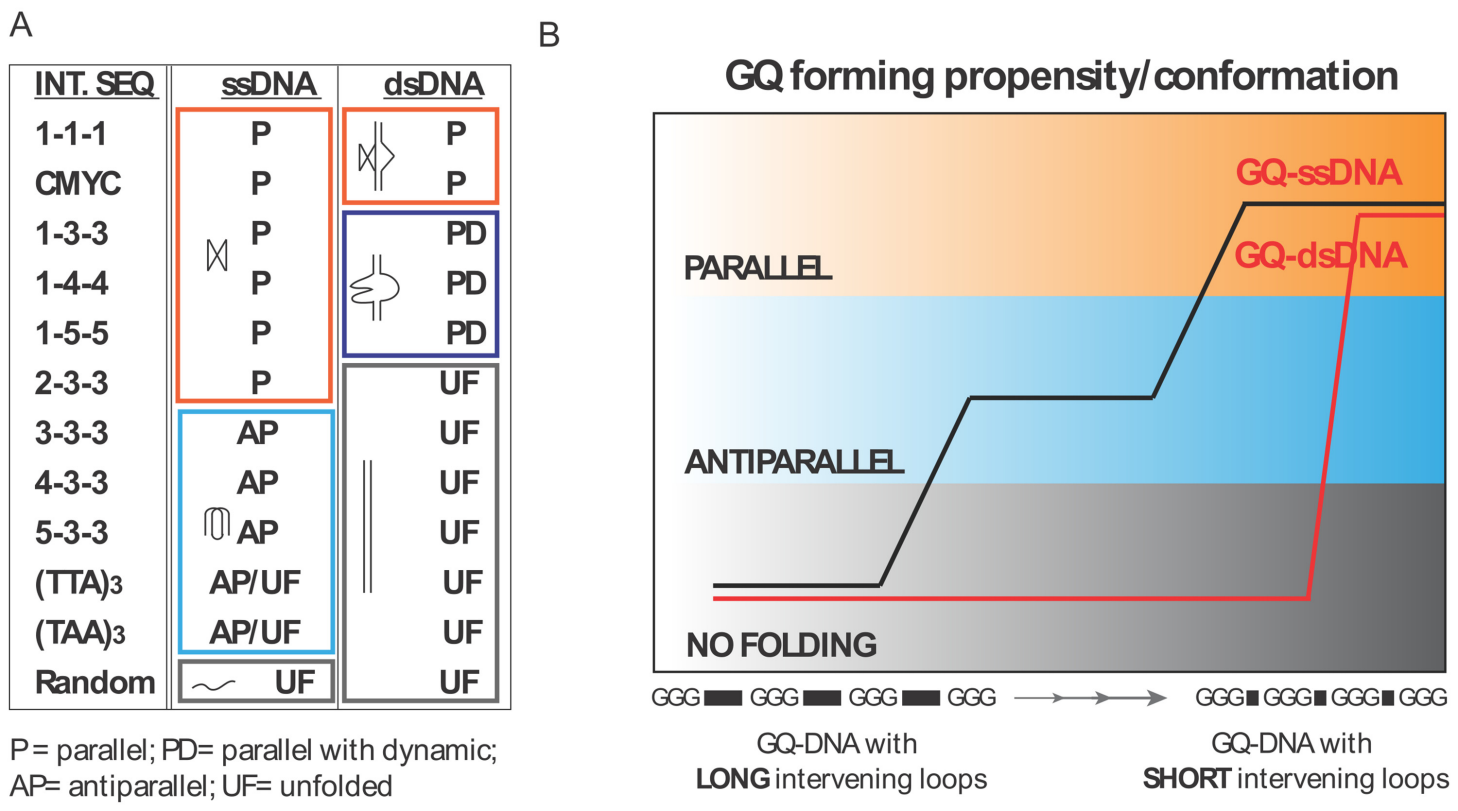
Summary of GQ-forming propensity. (**A, B**) The loop composition governs the GQ-forming potential differently in ssDNA and dsDNA.

Our data clearly show that the GQ with limited loop length can form in dsDNA in the presence of a molecular crowding condition (41). Interestingly, once folded, these structures stay folded for a long duration even after the removal of the molecular crowding reagent. Furthermore, increasing the loop length of these structures results in decreased NMM binding without increased CV binding, suggesting that in dsDNA, lengthening the loop does not induce a transition to an antiparallel state. Our results reveal that the antiparallel folding may only exist in the context of ssDNA, and such folding is not supported in dsDNA (Figure 5). The sequences that form into an antiparallel conformation in ssDNA cannot be held as folded structures in duplex. It is likely that the folding (thermodynamic) stability of the antiparallel GQ is weaker than that of Watson–Crick base pairing. It is plausible that the weak antiparallel GQ can be supported if GQ binding proteins or chemical ligand stabilize such structures in cells.

The smFRET approach enabled a direct and quantitative analysis in determining the fraction of GQ folded (high FRET) and unfolded/dsDNA (low FRET) molecules. We removed the low-FRET background provided by donor-only (acceptor-photobleached) molecules, by selecting molecules that contained both FRET pair dyes. The folded GQ quantified by smFRET matched the result obtained by the NMM ensemble fluorescence assay, thus validating the ensemble approach as a quantitative GQ detection method in both ss- and dsDNA. In addition, the smFRET revealed dynamic fluctuations in GQ structures formed by 1–4–4 and 1–5–5 DNAs, indicating that these sequences are able to spring back and forth between less folded and more folded conformations. If such dynamic property is exhibited in genomic DNA, the GQ may act like a flexible switch that opens and closes to control replication or transcription activity.

Taken together, we established a reliable fluorescence assay that can be readily applied to any types of DNA sequence for assessing its GQ-forming propensity and conformational specificity. Based on our mapping, we conclude that the GQ-forming pattern is: (i) significantly different between ssDNA and dsDNA; (ii) governed by loop length distribution and (iii) predictable based on the sequence composition and DNA context (Figure 5A). The GQ-forming potential outlined for ssDNA and dsDNA here (Figure 5B) will serve as a useful index for future studies.

## Supplementary Material

SUPPLEMENTARY DATA
