# Supplementary material for: G-quadruplex formation in double strand DNA probed by NMM and CV fluorescence
Source: Nucleic Acids Res. 2015 Jul 21;43(16):7961–70. doi: 10.1093/nar/gkv749 (PMC4652765; doi:10.1093/nar/gkv749)
Supplement: SUPPLEMENTARY DATA [file supp_43_16_7961__index.html]

G-quadruplex formation in double strand DNA probed by NMM and CV fluorescence — G-quadruplex formation in double strand DNA probed by NMM and CV fluorescence — SUPPLEMENTARY DATA 

# G-quadruplex formation in double strand DNA probed by NMM and CV fluorescence

## SUPPLEMENTARY DATA

- SUPPLEMENTARY DATA
