# Supplementary material for: G-quadruplex formation in double strand DNA probed by NMM and CV fluorescence
Source: Nucleic Acids Res. 2015 Jul 21;43(16):7961–70. doi: 10.1093/nar/gkv749 (PMC4652765; doi:10.1093/nar/gkv749)

**SUPPLEMENTARY INFORMATION**

**G-quadruplex Formation in Double Strand DNA probed by NMM and CV Fluorescence**

Alex Kreig^1^, Jacob Calvert^1^, Janet Sanoica^1^, Emily Cullum^1^, Ramreddy Tipanna^1^, and Sua Myong^1,2,3,4^

1. Bioengineering Department, University of Illinois; 1304 W. Springfield Ave. Urbana, Illinois 61801.
2. Biophysics and Computational Biology; 1110 W. Green St. Urbana, Illinois 61801
3. Institute for Genomic Biology; 1206 Gregory Drive, Urbana, Illinois 61801
4. Physics Frontier Center (Center of Physics for Living Cells), University of Illinois; 1110 W. Green St. Urbana, Illinois 61801

Corresponding author: Sua Myong [smyong@illinois.edu](mailto:smyong@illinois.edu)

Supplementary Figure 1

Supplementary Figure 2

Supplementary Figure 3

Supplementary Figure 4

Supplementary Figure 5

Table S1

SUPPLEMENTARY FIGURE 1


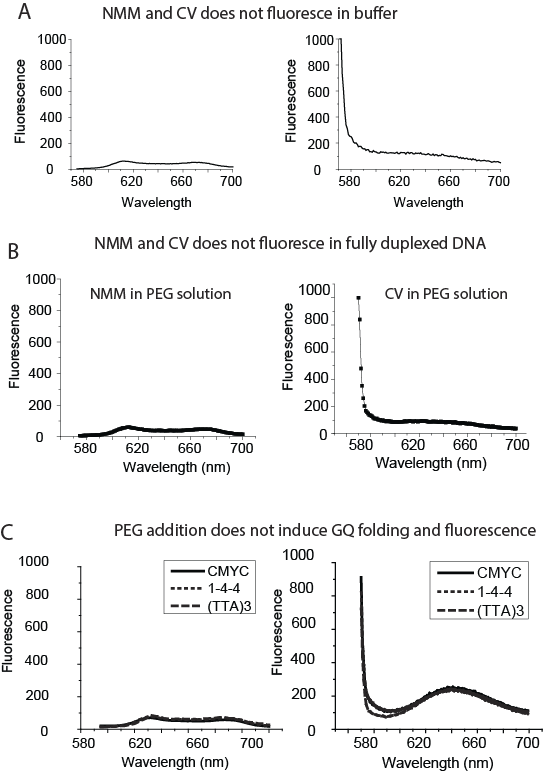


**Figure S1 PEG effect on GQ measurement** (A) NMM and CV shows no fluorescence in the imaging buffer which contains 4% PEG. (B) When NMM and CV are applied to fully duplexed DNA prepared by annealing in the absence of PEG, there is no fluorescence of both ligands (C) PEG added after DNA annealing in the absence of PEG does not induce GQ folding, hence no NMM and CV fluorescence.

SUPPLEMENTARY FIGURE 2


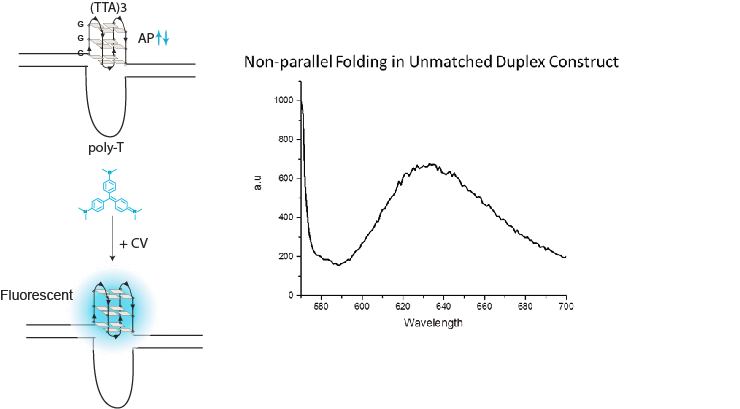


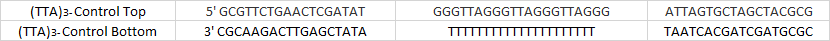


**Figure S2** **CV fluorescence within a unmatched non-parallel GQ within a duplex construct** The CV fluorescence for the GQ (TTA)3 is seen only when it shows an inability to duplex with the opposite stand. In this case, the opposite strand was composed of poly-T’s.

SUPPLEMENTARY FIGURE 3


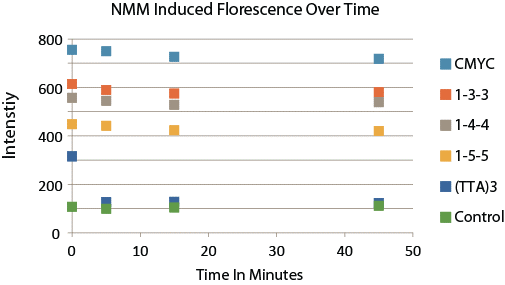


**Figure S3** **Stability of NMM fluorescence over time** The NMM fluorescence for CMYC, 1-3-3, 1-4-4, 1-5-5, (TTA)3 and control DNA was maintained at a stable intensity value over 45 minutes.

SUPPLEMENTARY FIGURE 4

**Figure S4** **CD cannot measure GQ formation in duplex DNA** The CD spectra for PEG in buffer, CMYC, 1-4-4, (TTA)3 exhibits a peak at 270 nm, which is different from the expected peak for parallel, antiparallel and mixed hybrid GQ conformation. Based on this data, CD cannot be used to measure GQ formation in duplexed DNA. All measurement were made in the standard buffer used for all other measurement (see method).

SUPPLEMENTARY FIGURE 5


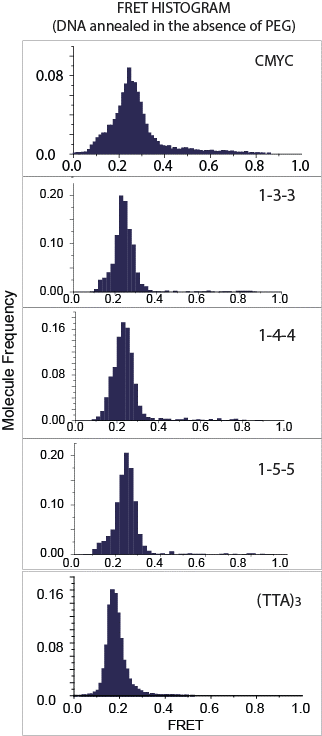


**Figure S5** **No GQ formation evidenced by low FRET** All dsDNA constructs, CMYC, 1-3-3, 1-4-4, 1-5-5 and (TTA)3 that were annealed in the absence of PEG displays low FRET, suggesting no formation of GQ.

Table S1


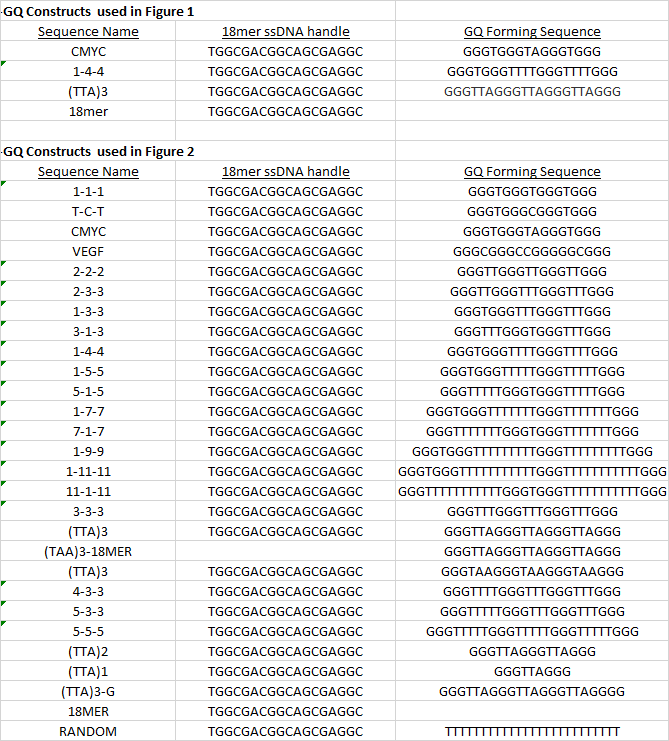


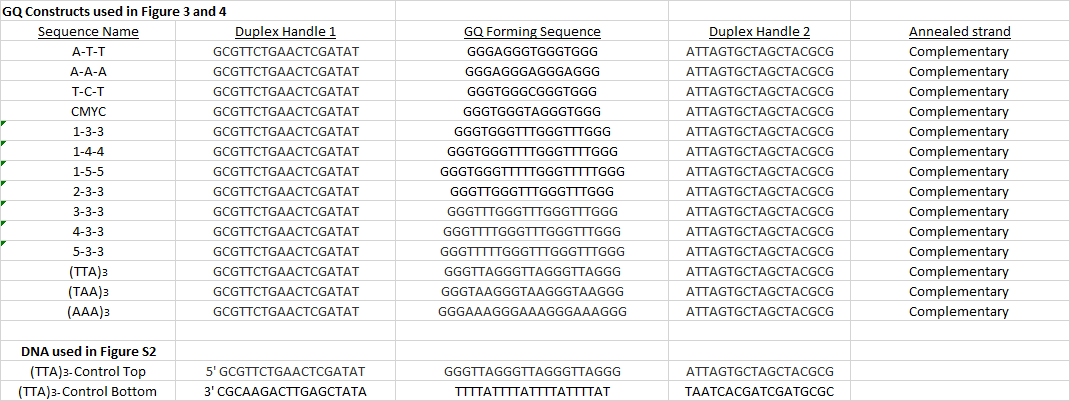

Supplement: SUPPLEMENTARY DATA [file supp_gkv749_nar-01009-f-2015-File007.docx]
